# Extracellular protonation modulates cell-cell interaction mechanics and tissue invasion in human melanoma cells

**DOI:** 10.1038/srep42369

**Published:** 2017-02-13

**Authors:** Verena Hofschröer, Alexander Koch, Florian Timo Ludwig, Peter Friedl, Hans Oberleithner, Christian Stock, Albrecht Schwab

**Affiliations:** 1 Institute of Physiology II, University of Münster, Münster, Germany; 2 Radboud University Medical Centre, Radboud Institute for Molecular Life Sciences, Department of Cell Biology, Nijmegen, The Netherlands; 3 David H. Koch Center for Applied Research of Genitourinary Cancers, The University of Texas MD Anderson Cancer Center, Houston, Texas, United States; 4 Cancer Genomics Center, CG Utrecht, The Netherlands; 5 Department of Gastroenterology, Hannover Medical School, Hannover, Germany

## Abstract

Detachment of cells from the primary tumour precedes metastatic progression by facilitating cell release into the tissue. Solid tumours exhibit altered pH homeostasis with extracellular acidification. In human melanoma, the Na^+^/H^+^ exchanger NHE1 is an important modifier of the tumour nanoenvironment. Here we tested the modulation of cell-cell-adhesion by extracellular pH and NHE1. MV3 tumour spheroids embedded in a collagen matrix unravelled the efficacy of cell-cell contact loosening and 3D emigration into an environment mimicking physiological confinement. Adhesive interaction strength between individual MV3 cells was quantified using atomic force microscopy and validated by multicellular aggregation assays. Extracellular acidification from pH_e_7.4 to 6.4 decreases cell migration and invasion but increases single cell detachment from the spheroids. Acidification and NHE1 overexpression both reduce cell-cell adhesion strength, indicated by reduced maximum pulling forces and adhesion energies. Multicellular aggregation and spheroid formation are strongly impaired under acidification or NHE1 overexpression. We show a clear dependence of melanoma cell-cell adhesion on pH_e_ and NHE1 as a modulator. These effects are opposite to cell-matrix interactions that are strengthened by protons extruded via NHE1. We conclude that these opposite effects of NHE1 act synergistically during the metastatic cascade.

Melanoma arises from the malignant transformation of melanocytes located in the stratum basale of the epidermal skin. Melanomas are the most aggressive skin cancers accounting for 80% of skin cancer induced deaths. As in nearly all forms of cancer, the formation of metastases is crucial for patient prognosis. Once metastasised the 5-year survival rate of melanoma patients drops to only 14%^1^,^2^. Adequate prognostic markers are missing and effective treatment possibilities have been lacking so far^2^. Currently, immunotherapy strategies provide new hopes in the treatment of advanced melanoma^3^.

An early step in the so-called metastatic cascade is the detachment of individual cells or cell clusters from the primary tumour. This is followed by migration of cancer cells through the extracellular matrix, intravasation, circulation and survival in lymph and blood vessels, adhesion to endothelial cells and extravasation out of the vascular system^4^. Melanoma cells escape the “control” of surrounding keratinocytes among others through (i) down-regulation of E-cadherin which mediates adhesion to keratinocytes, (ii) up-regulation of MCAM which can underlie melanoma-melanoma and/or melanoma-fibroblast interaction and (iii) loss of basement membrane anchorage through altered expression of integrins^5^. Preventing initial cell detachment from the primary tumour could therefore be a strategy to diminish melanoma metastasis.

High metabolic activity and limited diffusion lead to hypoxia in fast growing tumours. The concomitant anaerobic metabolism increases the intracellular acid load. Protons are extruded by the cells leading to the typical extracellular acidification. Thus, the gradient from the extracellular pH (pH_e_) to intracellular pH (pH_i_) may even be reversed so that pH_e_ of solid tumours is more acidic than pH_i_ and may be as low as pH_e_6.7^6^,^7^,^8^. In order to compensate for this altered pH homeostasis, acid-extruding transporters are upregulated and/or highly active in many forms of cancer to maintain pH_i_^9^. One of these transporters located in the plasma membrane is the Na^+^/H^+^ exchanger isoform 1 (NHE1) which imports Na^+^ and exports H^+^. It thereby contributes to an extracellular acidosis and was already described to be constitutively active in tumour cells^10^,^11^. Both, NHE1 activity and/or NHE1 expression may be increased in tumour cells among others because of dysregulation of its C-terminus^12^,^13^, because of mutations of tumour suppressors such as merlin or because of the local acidosis^14^. In migrating human melanoma cells, NHE1 is not homogeneously expressed but concentrates at the leading edge of the lamellipodium^15^,^16^. Hence, the proton concentration varies at the outer surface of the plasma membrane with relatively acidic pH values (pH_e_6.95) at the leading edge and more alkaline values (pH_e_7.15) at the rear end of polarised cells^15^,^17^. This pH_e_ gradient is preserved by the glycocalyx^18^.

Previously, we had shown that melanoma cell migration strongly depends on pH_e_ and NHE1 activity. It is inhibited by extracellular acidification below pH_e_7.0 and/or NHE1 inhibition^15^,^19^. Mechanistically, this could be related to a concentration of NHE1 at sites of focal adhesion at the front of migrating melanoma cells^20^ and a marked pH sensitivity of α_2_β_1_ integrins^19^,^21^. By producing a localised acidification at sites of focal adhesion NHE1 promotes the formation of integrin-collagen I bonds at the front. Its absence at the rear, in turn, facilitates the cell detachment from the underlying matrix. The impact of NHE1 on cell-matrix adhesion may be further modified by carbonic anhydrase IX, another tumour-associated pH-regulatory transmembrane enzyme that also localises to focal adhesion structures^22^. Moreover, CA IX was shown to modulate cell-cell contacts via an E-cadherin-dependent interaction with β-catenin^23^. Studies on the closure of chronic skin wounds revealed that pH_e_ gradients decrease migration, viability and proliferation of keratinocytes at the wound periphery during healing. Interestingly, NHE1 was predominantly expressed at the wound periphery, where low pH_e_ values occur, providing an explanation of how NHE1 could contribute to centrifugal pH_e_-gradients in chronic wounds^24^. In this context it is notable that NHE1 expressed in keratinocytes also contributes to the acid pH_e_ physiologically found in superficial layers of the skin^25^.

The above cited studies suggest that an acidic pH_e_ in the tumour microenvironment is likely to play an important role in different steps of the metastatic cascade. Based on this knowledge and the fact that upregulation of NHE1 has been correlated with tumour malignancy and NHE1 function through increased proton efflux with tumour cell invasiveness^26^, we hypothesised that NHE1 affects cell-cell adhesion in human melanoma. We adopted a three-dimensional (3D) model in order to mimic the complex tumour nanoenvironment and physical constraints of a collagen matrix more closely. Interestingly, melanoma cells detach more easily from the primary spheroid in an acidic environment. Using single cell force spectroscopy (SCFS) with atomic force microscopy (AFM) we found a reduction of cell-cell-adhesion forces upon extracellular acidification and NHE1 overexpression.

## Results

### In a 3D assay, acidification affects cell migration and adhesion

**Figure 1:**
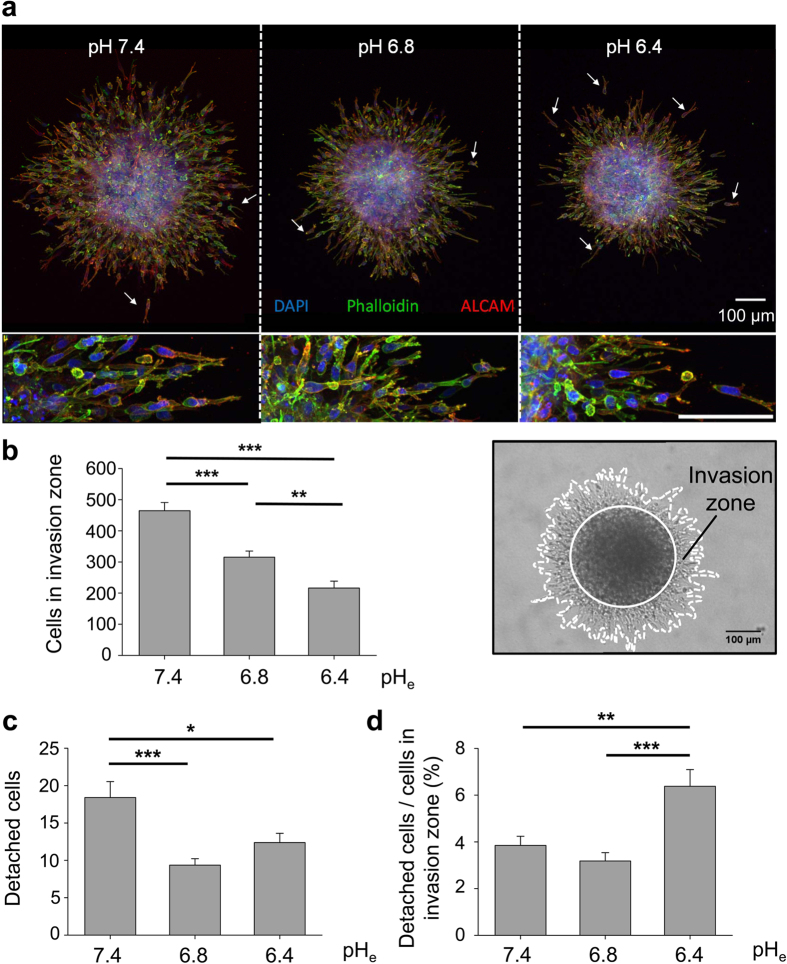
3D emigration assays through a collagen I matrix. **(a)** Maximum projection images of z-stacks obtained by confocal laser scanning microscopy reveal that acidification (i) controls melanoma cell migration by lowering the area of invasion and (ii) increases the number of detached cells (white arrows) after 24 h. Activated leukocyte cell adhesion molecule (ALCAM) is expressed in all three conditions. Scale bar = 100 μm in images of higher magnification in the second row. **(b)** Quantification of the number of cells that migrate into the collagen mesh and form the invasion zone around the initial spheroid. The invasion zone is calculated as the difference of the total area of the spheroid (dashed white line) and the area of the spheroid core (solid white circle). Extracellular acidification decreases the absolute number of cells in the invasion zone (pH_e_7.4: 464.6 ± 26.1 cells (N = 5 experiments with n = 20 spheroids); pH_e_6.8: 315.3 ± 19.6 cells (N = 4, n = 19); pH_e_6.4: 216.4 ± 21.9 cells (N = 5, n = 24)). **(c)** Absolute number of cells that detach from the initial spheroid (pH_e_7.4: 18.4 ± 2.1 cells; pH_e_6.8 and pH_e_6.4 were 9.4 ± 0.9 and 12.4 ± 1.2 cells). **(d)** Number of detached cells normalised to the total number of cells in the invasion zone. Most cells detach at the lowest pH_e_ value of 6.4 (pH_e_7.4: 3.85 ± 0.39%; pH_e_6.8: 3.18 ± 0.35%; pH_e_ 6.4: 6.38 ± 0.72%). Statistical significance was observed by one-way ANOVA followed by student’s t-test (parametric data).

In a first set of experiments we determined pH_e_-dependent migration and adhesion patterns in a 3D extracellular collagen I matrix. Using the hanging-drop method, MV3 cells formed stable multicellular spheroids after 36 h in culture. MV3 cells were allowed to emigrate from multicellular spheroids into a rat tail collagen I matrix mainly showing mesenchymal migration mode unaffected by changes in pH_e_ (Fig. 1a, close-ups shown in supplementary Fig. S1). Lowering pH_e_ from 7.4 to pH_e_6.8 and pH_e_6.4 progressively reduced the total number of emigrated cells within the invasion zone (Fig. 1b). The absolute number of invading cells beyond the original spheroid margin (referred to as ‘detached cells’; Fig. 1c) was also reduced by half when pH_e_ was lowered from pH_e_7.4 to pH_e_6.4. However, the percentage of single cells detached from the spheroid, quantified as the number of separate cells beyond the spheroid margin and normalised to the total number of cells in the invasion zone, had doubled (Fig. 1d). Thus, despite moderate reduction of cell migration speed, the cell subset retaining persistent invasion capability developed near-exclusive single-cell migration.

### Extracellular acidification lowers the strength of cell-cell adhesion

**Figure 2:**
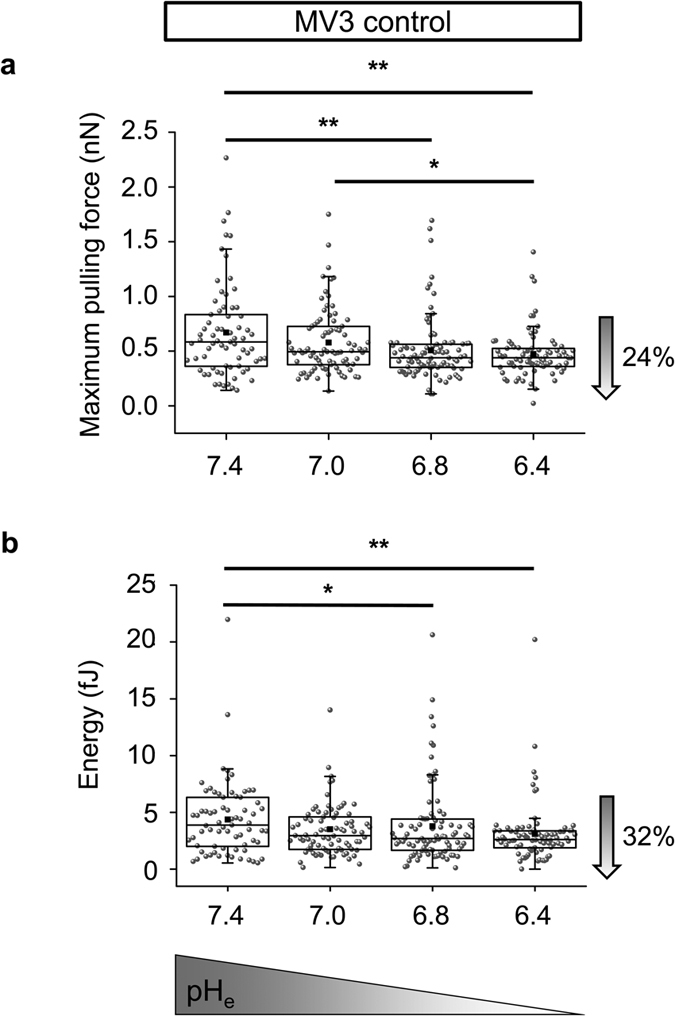
Single cell force spectroscopy at varying pH_e_. Extracellular acidification progressively lowers the strength of cell-cell adhesion in MV3 control cells as indicated by a decline of the **(a)** maximum pulling force (pH_e_7.4: 0.58 nN (0.36/0.83 nN, N = 4 cells attached to the cantilever, probing n = 71 cells on the underlying matrix); pH_e_7.0: 0.49 nN (0.38/0.73 nN, N = 4, n = 83); pH_e_6.8: 0.44 nN (0.35/0.56 nN, N = 5, n = 91) and pH_e_6.4: 0.44 nN (0.36/0.53 nN, N = 4, n = 80)) and **(b)** the adhesive interaction energy (pH_e_7.4: 3.88 fJ (2.0/6.32 fJ); pH_e_7.0: 2.95 fJ (1.74/4.6 fJ); pH_e_6.8: 2.71 fJ (1.66/4.4 fJ) and pH_e_6.4: 2.62 fJ (1.91/3.38 fJ)). Paired experiments were carried out so that cell-cell interaction forces were measured for the same cells at different pH_e_ values. Statistical significance of the differences was assessed by Kruskal-Wallis ANOVA followed by the Mann-Whitney U test.

To gain further insight into the mechanisms underlying pH_e_-dependent cell-cell detachment, AFM-based SCFS was applied. To this end MV3 cells were seeded on a 2D collagen I matrix and another cell of the same kind was attached to the cantilever and lowered onto the underlying adherent cells. The forces needed to retract the cantilever were measured. We performed paired experiments and tested consecutively the impact of different pH_e_ values on cell-cell adhesion for each cell attached to the cantilever. The results are summarised in Fig. 2. Using MV3 empty vector cells the adhesive interaction forces decreased by increasing the proton concentration in the surrounding medium as shown in Fig. 2a. Thus, the maximum pulling force was reduced by 24% when pH_e_ was lowered from pH_e_7.4 to pH_e_6.4. The pH-dependence of the adhesion energy, i.e. the work that is required to detach cells from each other, was even more pronounced and lowered by 32% (Fig. 2b).

### Cell-cell adhesion force is low in NHE1-overexpressing cells and high in NHE1-deficient cells

**Figure 3:**
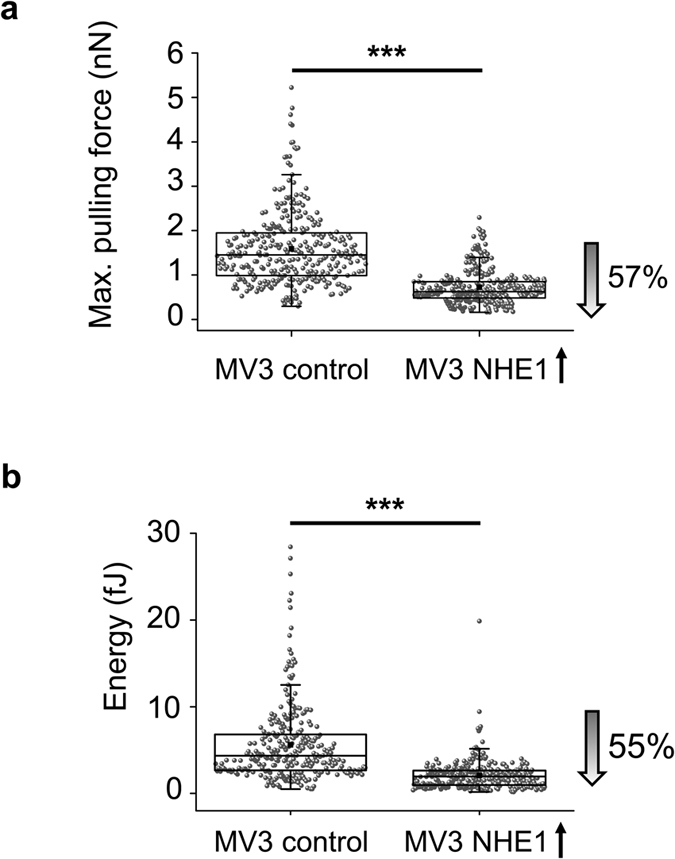
Single cell force spectroscopy using MV3 cells with different NHE1 expression levels. AFM experiments reveal that the cell-cell adhesion force, represented by the **(a)** maximum pulling force and the **(b)** adhesion energy, is lower in NHE1-overexpressing cells (0.62 nN (0.48/0.85 nN), N = 9 cells attached to the cantilever probing n = 354 cells on the underlying matrix; 1.96 fJ (0.97/2.65 fJ), N = 9, n = 315) than in MV3 control cells (1.45 nN (0.99/1.95 nN), N = 8, n = 326; 4.35 fJ (2.67/6.8 fJ), N = 8, n = 281). Statistical significance of the differences was assessed by the Mann-Whitney U test.

We next tested whether the acid extruding NHE1 not only affects cell migration^15^,^19^ but also cell-cell adhesion. Therefore, we compared MV3 cells overexpressing NHE1 and NHE1-deficient cells. The maximum pulling force necessary to separate two individual melanoma vector control cells was 1.45 nN, the adhesion energy was 4.35 fJ. (Fig. 3). In contrast, the maximum pulling force was 57% lower in MV3 cells overexpressing NHE1 and the adhesion energy was reduced by 55%. Moreover, NHE1-deficient MV3 cells showed a significant 12% increase in the maximum pulling force compared to the respective control (supplementary Fig. S2). Thus, because increased NHE1 expression is known to lower pericellular pH_em_, these findings were in line with the above described AFM measurements with varying proton concentrations in the bathing solution.

### NHE1-overexpressing cells do not form stable tumour spheroids in multicellular cell aggregation assays

**Figure 4:**
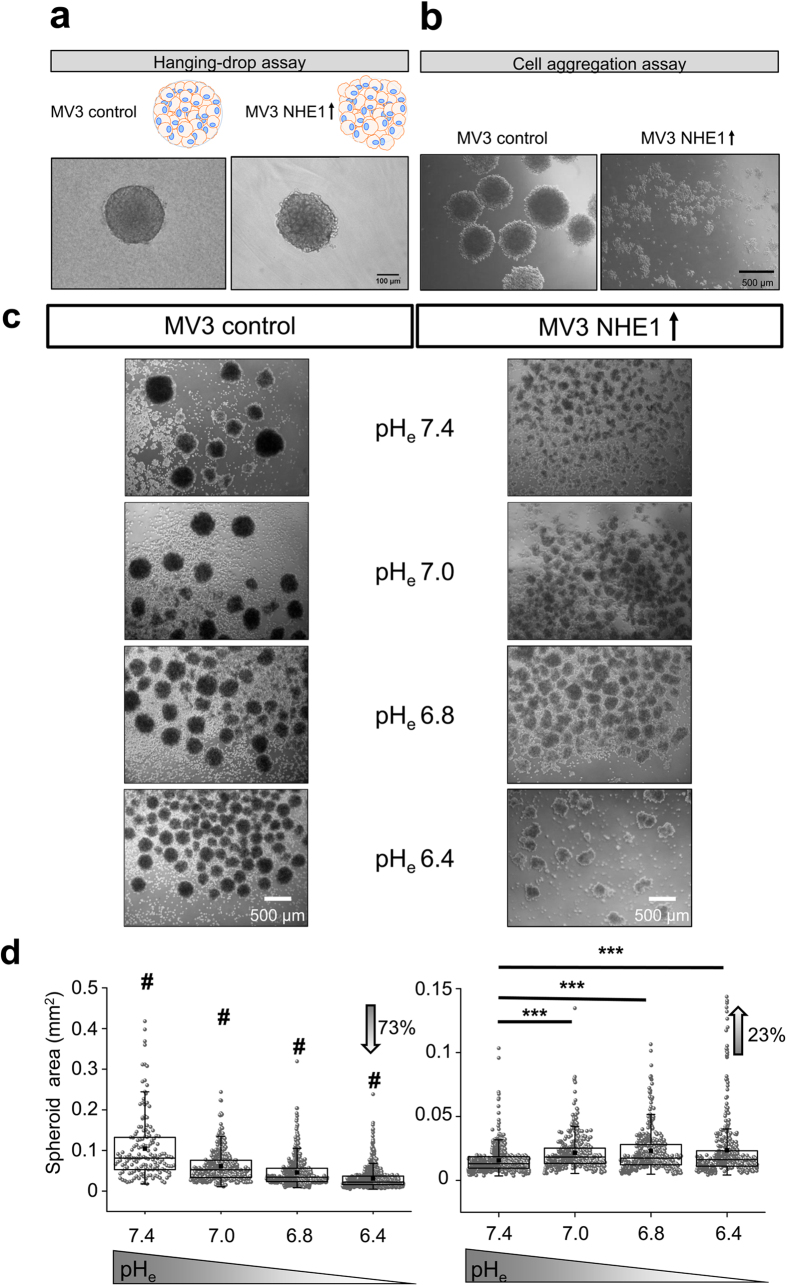
Multicellular adhesion assays. **(a)** Both MV3 control and MV3 NHE1-overexpressing cells form tumour spheroids using the hanging-drop assay that is presented in Fig. 2a. However, spheroids of NHE1-overexpressing cells are less regular and circular. **(b)** For the cell aggregation assays, MV3 cells were incubated in experimental medium on a shaker overnight. Here, NHE1-overexpressing cells do not form stable tumour spheroids thus pointing towards weaker cell-cell adhesion. MV3 control cells formed spheroids with an average diameter of 466 μm (399/526 μm, N = 3 experiments, n = 84 spheroids) and a projected cross-sectional area of 0.16 mm^2^ (0.12/0.19 mm^2^, N = 3, n = 79). **(c,d) Left:** Increasing the extracellular proton concentration reduces the spheroid size of MV3 control cells (cross-sectional area detected by light microscopy: pH_e_7.4: 0.081 mm^2^ (0.053/0.013 mm^2^), N = 4, n = 154; pH_e_7.0: 0.052 mm^2^ (0.033/0.076 mm^2^), n = 356; pH_e_6.8: 0.034 mm^2^ (0.023/0.056 mm^2^), n = 517; pH_e_6.4: 0.022 mm^2^, (0.016/0.037 mm^2^), n = 890)) and increases the spheroid number. **Right:** MV3 NHE1-overexpressing cells form fewer cell aggregates at pH_e_7.4 than control cells. However, the adhesive strength between two cells slightly increases upon acidification as shown by a small rise of cell aggregate size. Quantification of pH_e_-dependent spheroid formation in MV3 NHE1-overexpression cells: pH_e_7.4: 0.013 mm^2^ (0.009/0.019 mm^2^), N = 3, n = 636; pH_e_7.0: 0.018 mm^2^ (0.013/0.025 mm^2^), n = 387; pH_e_6.8: 0.017 mm^2^ (0.012/0.028 mm^2^), n = 398; pH_e_6.4: 0.016 mm^2^ (0.011/0.023 mm^2^), n = 413. # = significant difference (p < 0.001) to all groups. Statistical significance was tested using Kruskal-Wallis ANOVA and Mann-Whitney U test.

So far, our results indicate that an extracellular acidification or increased NHE1 activity weakens cell-cell contacts. To further evaluate this concept we tested whether NHE1 also contributes to spheroid formation and thus cell-cell adhesion in a multicellular assay. In the hanging-drop method employed for the emigration assays, MV3 control cells and NHE1-overexpressing MV3 cells formed stable spheroids within 36 h (Fig. 4a). MV3 control cell spheroids were by and large round, whereas those of NHE1 overexpressing MV3 cells were irregularly shaped and more loosely connected pointing towards weaker cell-cell connections.

In addition, we performed cell aggregation assays to test the involvement of NHE1 and pH_e_ without supplementing the initial cell suspension with bovine collagen and methylcellulose for spheroid formation. MV3 control cells formed spheroids after ~16 h with an average diameter of 466 μm and a projected cross-sectional area of 0.16 mm^2^ as represented in Fig. 4b. NHE1 overexpression prevented spheroid formation which is consistent with the decreased adhesion observed in SCFS. Furthermore, extracellular acidification also reduced the spheroid size of MV3 control cells (Fig. 4c, left panel) in that the cross-sectional area decreased by 72.8% from pH_e_7.4 to pH_e_6.4 (quantification in Fig. 4d, left panel). Thus, not only 3D emigration out of the melanoma cell spheroid, but also their formation is dependent on pH_e_ and NHE1.

### Extracellular acidification increases adhesion between NHE1-overexpressing MV3 cells

**Figure 5:**
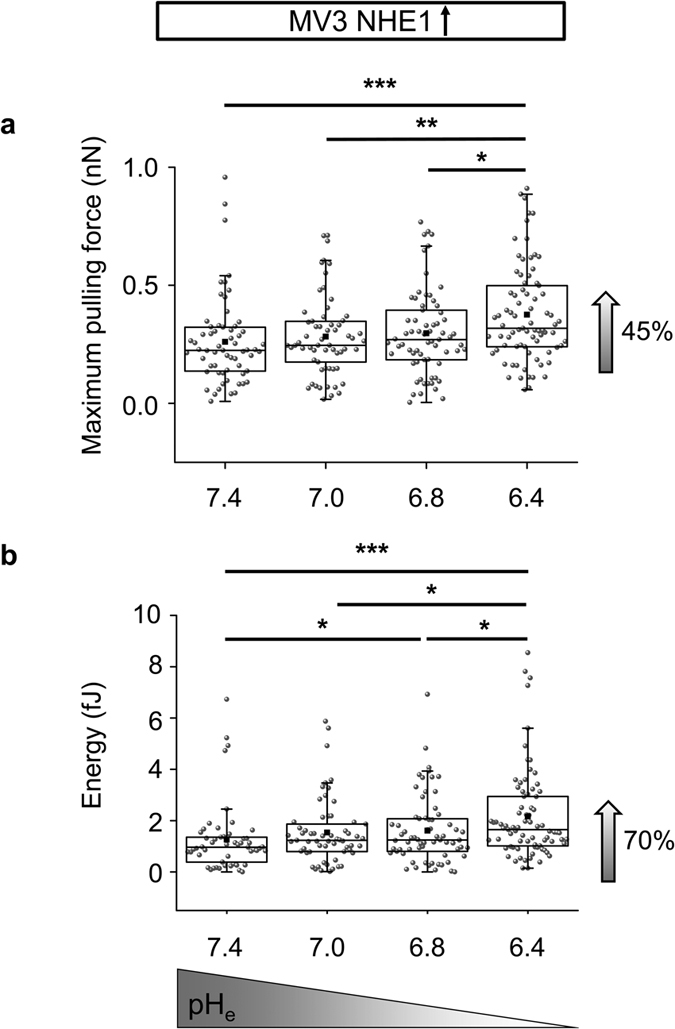
Single cell force spectroscopy using MV3 NHE1-overexpressing cells at varying pH_e_. **(a)** Maximum pulling force and **(b)** adhesion energy are increased in SCFS measurements upon acidification of NHE1-overexpressing cells (pH_e_7.4: 0.22 nN (0.14/0.32 nN) and 0.97 fJ (0.5/1.36 fJ), N = 5, n = 62; pH_e_7.0: 0.25 nN (0.17/0.35 nN) and 1.24 fJ (0.8/1.9 fJ), N = 5, n = 61; pH_e_6.8: 0.27 nN (0.18/0.39 nN) and 1.25 fJ (0.8/2.07 fJ), N = 5, n = 67; pH_e_6.4: 0.32 nN (0.24/0.5 nN) and 1.65 fJ (1.02/2.94 fJ), N = 5, n = 79). Force measurements were performed in paired experiments by exposing the same cells to different pH_e_ values. Statistical significance was tested using Kruskal-Wallis ANOVA and Mann-Whitney U test.

To combine both findings, namely that acidification of the extracellular environment as well as increased NHE1 expression lower cell-cell adhesion, NHE1-overexpressing cells were exposed to varying pH_e_. Surprisingly, melanoma cells formed larger spheroids in the multicellular approach upon increasing the extracellular proton concentration (Fig. 4c,d, right panel). We observed no additive effect of NHE1 expression and extracellular pH in paired SCFS experiments. At pH_e_7.4, NHE1 overexpressing cells showed the lowest cell-cell adhesion strength and energy observed in this study (0.22 nN (0.14/0.32 nN) maximum pulling force and 0.97 fJ (0.5/1.36 fJ) adhesion energy; Fig. 5a,b). Increasing the proton concentration enhanced the strength of cell-cell adhesion.

### NHE1 expression correlates with the expression of melanoma cell adhesion molecule

**Figure 6:**
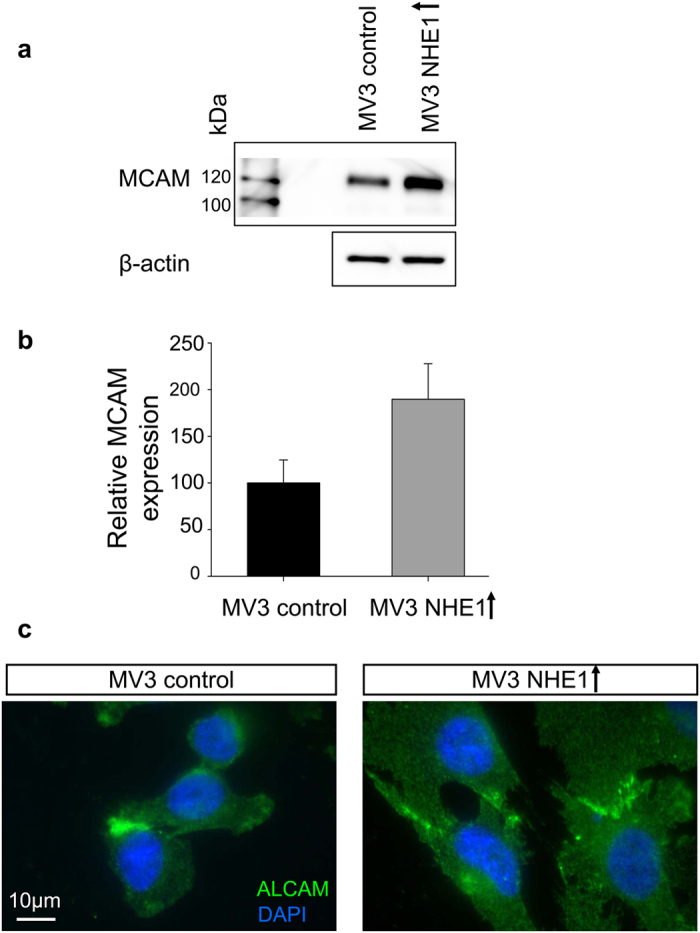
Expression of Cell Adhesion Molecules. **(a,b)** Melanoma cell adhesion molecule (MCAM) expression is higher in NHE1-overexpressing cells than in control cells (MV3 control: 100% ± 24.6%; MV3 NHE1 + : 189.7% ± 38.1). The relative expression of MCAM is corrected for β-actin. **(c)** Activated leukocyte cell adhesion molecule (ALCAM) concentrates at cell-cell contacts.

Western Blot analyses using MV3 cell clones with different NHE1 expression levels were performed. Whole protein was isolated following cell aggregation assays. Melanoma cell adhesion molecule (MCAM) correlated with NHE1 expression in that NHE1-overexpressing cells showed a 90% higher amount compared to control (Fig. 6a,b). Moreover, ALCAM expression was verified by immunofluorescence staining and microscopy on 2D cultures (3D ALCAM staining shown in Fig. 1a). In all four cell clones (data shown for NHE1-overexpressing cells and control cells in Fig. 6c), ALCAM was expressed and, importantly, detected at sites of cell-cell contacts.

## Discussion

Cell-cell interaction is an important characteristic of tumour cells: on the one hand, cells form a primary tumour and, on the other hand, they switch their behavior to loosen intercellular connections and initiate the process of migration and emigration. The present study aimed to elucidate aspects of the impact of the tumour nanoenvironment, at least partially mediated by NHE1, on cell-cell adhesion of human melanoma cells.

Metabolic reprogramming of tumour cells leads to an increased intracellular acid production. Transport proteins such as NHE1 effectively extrude these protons^10^ thereby contributing to the typical acidification of solid tumours. Their (over-)expression or activation correlates with the malignancy of tumour cells^10^,^11^,^27^ so that proteins involved in intra- and extracellular pH regulation are important candidates in regulating the metastatic behaviour of tumour cells. Their role in cell-matrix adhesion and migration of tumour cells has been reviewed previously in detail^28^,^29^. Both pH_e_ and NHE1 strongly affect cell adhesion to a collagen I matrix, cell migration and invasion in human melanoma cells. The pH nanoenvironment at the outer leaflet of the plasma membrane at focal adhesions has a major impact^17^. In the present study, cell migration was significantly reduced in a 3D model in the presence of increased extracellular proton concentration. Thereby, we could recapitulate previous studies investigating migration of single melanoma cells and of keratinocytes during wound healing^15^,^19^,^24^.

SCFS provides data of mechanical forces that prevail between living melanoma cells. Adhesion forces might serve as an indicator of tumour cell invasiveness since cells with low adhesion forces could be more likely to separate from each other. Modes of single cell migration are defined by a lack of cell-cell adhesion through loss of long-lasting adhesive junctions^30^,^31^. We observed that an extracellular acidification induces higher detachment rates of single cells in the 3D emigration assay and lowered cell-cell adhesion strength in the SCFS analysis. Preliminary data (supplementary Fig. S3) indicate that this phenomenon is not only restricted to melanoma cells but that decreased cell-cell adhesion through extracellular acidification is also found in other forms of cancer. 4T1 breast cancer cells show multicellular strains at pH_e_7.4 and single cell behaviour at acidic pH_e_ values of 6.8 and 6.4. While cell migration is clearly impaired in an acidic nanoenvironment, the facilitated detachment of single melanoma cells from the spheroids could point to an alternative and adaptive mechanism to effectively start the metastatic cascade even under adverse nanoenvironmental conditions. While the extracellular metabolic challenge due to acidification compromises the overall migration efficacy, likely through increased cell-matrix adhesion^19^, cell-cell adhesion is weakened, resulting in prominent individualisation of cells that eventually evade the perturbed site.

At first sight it is surprising to note that MV3 control and NHE1-overexpressing cells behave contradictorily with respect to the pH dependence of cell-cell adhesion. However, it should be kept in mind that pH_e_ and NHE1 are not equally distributed throughout tumour tissue. At the outer borders of a primary tumour, cells have sufficient access to blood vessels and pH values are more alkaline than in its centre. In human colon carcinoma spheroids (~300 μm diameter), pH_e_ values of ~6.9 in the core and ~7.45 at the outer borders were measured^32^. NHE1 expression is increased inside the tumour tissue compared to the extra-tumoural compartment. Furthermore, NHE1 expression also varies inside the tumour tissue in that it is highest (i) in peripheral and well-perfused regions of the tumour tissue^33^ and, more precisely, (ii) directly at the inner rim of the tumour (shown for C6 gliomas in a rat brain^33^). Putting the present data into a (patho-) physiological context by taking the distribution of pH_e_ and NHE1 into account, cells at the edge of the tumour tissue (represented by NHE1-overexpressing cells at pH_e_7.4) exhibit the lowest cell-cell adhesion and likely a high risk for detachment of cells as a starting point in the metastatic behaviour.

The question remains which adhesion molecules are involved in this model. NHE1 itself could contribute through differences in glycosylation or indirectly by affecting the glycocalyx. Protons extruded by NHE1 could neutralise the negatively charged glycocalyx and thereby modify its function. For carbonic anhydrase IX a modulation of cell-cell contacts via E-cadherin and beta-catenin was described^23^ and, furthermore, E-cadherin as well as N-cadherin binding are weakened by an acidic extracellular pH^34^,^35^. However, MV3 cells do not express multiple cadherins as previously determined by immunofluorescence analysis^36^ and also confirmed by RT-PCR analysis for E- and N-cadherin in this study (data not shown). Furthermore, 3D immunofluorescence detection of p120-catenin, a cytoskeletal adaptor for cadherins^37^,^38^, reveals a cytosolic localization rather than one at the plasma membrane (data not shown). On the other hand, the expression of cell-cell adhesion receptors of the immunoglobulin superfamily of cell adhesion molecules (CAMs) is higher in melanoma cells than in melanocytes^5^. ALCAM (CD 166) and MCAM (CD 146) were already described as intercellular adhesion molecules in melanoma cells and their expression correlates with enhanced melanoma development, metastatic properties and tumour progression (ALCAM^39^,^40^; MCAM^41^,^42^). ALCAM expression and localisation at cell-cell contacts was confirmed by immunofluorescence staining in all four of the used MV3 cell clones. Previously, ALCAM was reported to regulate adherens junctions in uveal melanoma cells whereas ALCAM-mediated invasiveness depends on the type of cadherin adhesion complexes^43^. Interestingly, expression of MCAM is NHE1-dependent: NHE1-overexpressing cells, showing less cell-cell adhesion, exhibit increased MCAM levels. In mice, CD146 is found to promote melanoma metastasis to the lungs whereas melanoma growth or tumour angiogenesis are not affected^44^. Another study reveals disrupted spheroid formation, less cell-cell adhesion and cell invasion *in vitro* using a blocking antibody against MCAM^45^. So far, our results do not allow to conclude that pH-dependent cell-cell adhesion is mediated by MCAM or ALCAM. Although MCAM is found at cell-cell contacts, its increased expression in NHE1-overexpressing cells is inconsistent with lower cell-cell adhesion in these cells. Further experiments are clearly needed to determine the roles of MCAM and ALCAM in pH-dependent cell-cell adhesion.

## Conclusion

**Figure 7:**
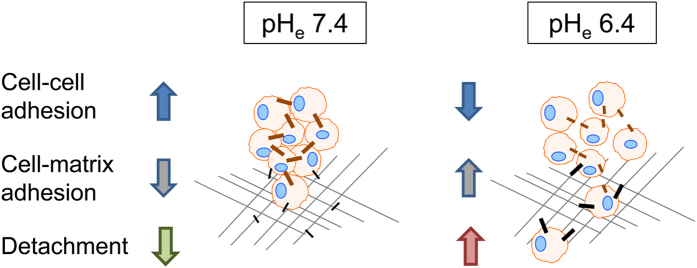
Summary. Extracellular acidification reduces cell-cell adhesion, while at the same time cell-matrix interaction is promoted. These effects might ease detachment of single cells from a primary tumour, the invasion into the surrounding tissue and thereby synergistically promote metastasis.

pH_e_ and NHE1 not only affect the cellular process of emigration but also intercellular adhesive bond strength and initial adhesion to form stable tumour spheroids. In summary, low cell-cell adhesion forces determined via SCFS indicate facilitated detachment of cells from the primary spheroid. NHE1 anchoring in the plasma membrane as well as acidification of the extracellular medium both reduce the strength of cell-cell contacts in melanoma. Interestingly, the pH-dependence of cell-cell adhesion strength is opposite to cell-matrix adhesion^19^ which is enforced by an extracellular acidification because of increased avidity of cell surface integrin receptors^21^. Hence, a synergistic interplay between cell-matrix and cell-cell adhesion seems to be in place to control the balance between different steps of the metastatic cascade. Mesenchymal invasion, as shown by MV3 cells, was described to be characterised by high cell-matrix adhesion and single cell migration was characterised by low cell-cell adhesion^30^. We therefore propose that NHE1 can promote metastasis by first facilitating cell detachment from the primary tumour and then modulating cell-matrix interaction to promote cell migration and invasion (Fig. 7). Thus, in combination with other strategies, targeting proton export may serve as a potential therapeutic strategy to at least partly overcome current treatment deficiencies in human melanoma.

## Methods

### Cell culture

Experiments were performed on the human melanoma cell line MV3 which originates from a malignant melanoma metastasis^46^. Cells were cultured at 37 °C and 5% CO_2_ in RPMI-1640 cell culture medium (with NaHCO_3_ and L-glutamine; Sigma-Aldrich, Taufkirchen, Germany) supplemented with 10% fetal calf serum (FCS, PAA Laboratories, Pasching, Austria).

To study possible effects of NHE1 on cell adhesion, we used two established cell clones that have been characterized previously: (i) NHE1-overexpressing MV3 cells^14^ and (ii) an NHE1-deficient MV3 cell clone^15^ with the respective controls. The degree of NHE1-overexpression corresponds to that induced by prolonged incubation in acidic (pH6.8) culture medium. NHE1-overexpressing cells were compared with cells transfected with the expression vector pcDNA3 (MV3 vector control). The NHE1-deficient cell clone was rescued by transfection with pcDNA-NHE1. Geneticin (G-418, 600 mg/l final concentration; Sigma) was added to the culture medium of transfected cells.

### 3D spheroid culture using the hanging-drop method

Multicellular tumour spheroids were formed with the hanging-drop method as previously described^31^,^47^. Briefly, MV3 cells were detached with EDTA (1 mmol/L) and resuspended in RPMI cell culture medium. 5.000 cells per drop were incubated upside-down in 30 μl droplets of medium containing methylcellulose (40%, Sigma-Aldrich) and bovine collagen (6.3 μg/ml, PureCol^®^; Advanced BioMatrix, San Diego, USA) for 36 h in a wet chamber (1x PBS) in a humidified 5% CO_2_ incubator (37 °C).

### Spheroid embedding in 3D matrix and fixation

MV3 vector control and MV3 NHE1-overexpressing spheroids were harvested and gently washed three times in PBS. Next, spheroids were mixed with a collagen solution on ice containing: acid-solubilised rat-tail collagen type I (5 mg/ml final concentration, Corning, New York, USA), PBS, 1N NaOH and HEPES (20 mmol/L) for maintaining pH 7.5 during polymerisation. Drop-gels were generated using 100 μl from the collagen solution per drop containing maximally 5 individual spheroids. Collagen lattices polymerised at 37 °C while the plate was turned every 60 sec to allow a central position of the spheroids in the z-plane ensuring 3D invasion. After ~20 min, bicarbonate-free RPMI-1640 medium supplemented with HEPES (10 mmol/L) and adjusted for the respective experimental pH values was added to the spheroid-containing collagen gels. After an experimental period of 24 h spheroids were fixed for 20 min at 37 °C in 4% paraformaldehyde in 0.2 mol/L sodium phosphate buffer (PB).

### 3D Immunofluorescence staining and confocal microscopy

Spheroids were blocked for ~8 h and stained in 0.1% BSA-PBS. For ALCAM staining, the primary antibody was incubated 1:70 overnight at 4 °C (mouse-anti ALCAM mAb, AZN-L50, IgG2A, Department of Tumour Immunology, Radboud Institute for Molecular Life Sciences, The Netherlands 47 2 mg/ml). After washing seven times in PBS for 1 h each, the secondary antibody (1:200, Alexa-Fluor-647-conjugated goat anti-mouse IgG; Invitrogen, Life Technologies, Carlsbad, USA) was incubated with DAPI (1:1000, Roche, Basel, Switzerland) and Alexa-Fluor-488-conjugated Phalloidin (1:200, Invitrogen) at 4 °C overnight. Samples incubated with just a secondary mouse IgG-antibody (Invitrogen) were run in parallel to the experiments for negative controls. After final washing, spheroids were imaged using a Leica TCS SP8 confocal microscope equipped with a dry 10x/0.4 NA air objective. Z-stacks were obtained at 5 μm slice intervals. Maximum projections were used to analyse and quantify migration and adhesion patterns using Fiji/Image J software (version 1.50 g). The invasion zone was defined as the total spheroid area after 24 h minus the area of the spheroid core (as shown for a light microscopy image for MV3 control cells at pH_e_ 6.8 in Fig. 1b). The number of cells was counted manually and DAPI staining was used to quantify cells that had migrated into the invasion zone. Detached cells were defined as those cells that had migrated beyond the original spheroid margin and lost contact to surrounding cells in x-, y- and z-dimension. N = individual experiments; n = spheroids.

### Single cell force spectroscopy (SCFS)

**Figure 8:**
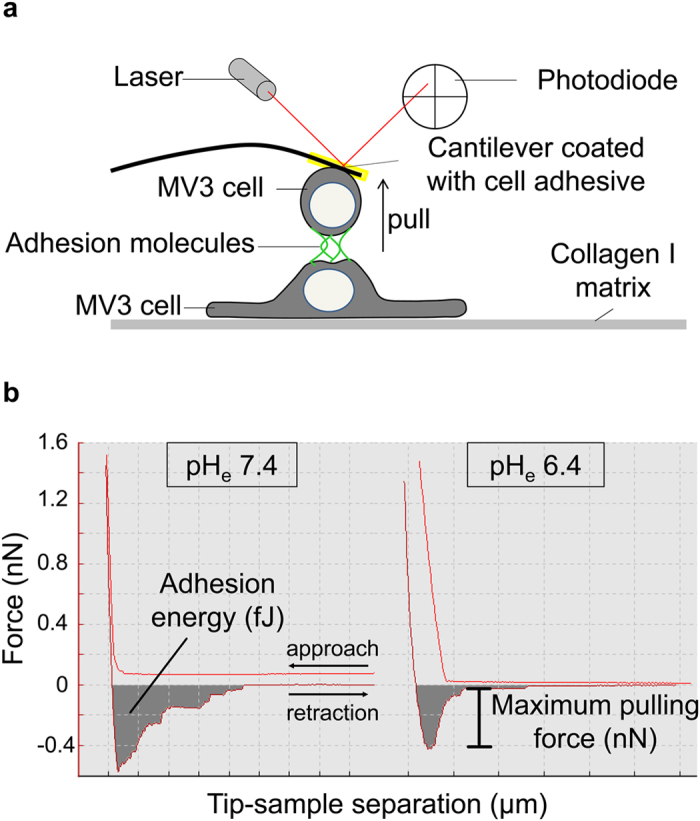
Schematic illustration of cell-cell adhesion analysis using AFM. **(a)** Single cell force spectroscopy. A single melanoma cell (MV3) attached to a flexible cantilever is brought into contact with another adherent melanoma cell of the same kind seeded on collagen I. When lowering the cantilever (approach curve), a defined force of 1.5 nN is applied to bring the cells into contact. After a contact time of 2 seconds, the cells are mechanically separated by retraction of the cantilever in z direction (retraction curve). **(b)** Data analysis. Representative force-distance curves for pH_e_7.4 and pH_e_6.4 illustrate the data analysis: the required adhesion energy is calculated from the area under the curve. The maximum pulling force needed to separate two individual melanoma cells is calculated from the lowest turning point of the retraction curve.

The absolute mechanical strength of cell-cell adhesion between melanoma cells was determined by means of SCFS using atomic force microscopy (AFM; Fig. 8a; CellHesion^®^ 200 module, JPK, Berlin, Germany)^48^. This AFM offers an enlarged z-piezo range of >110 μm so that the cantilever can be lifted high enough in order to separate two individual cells from each other. Deflection sensitivity and spring constant of the cantilever were determined prior to each experiment. Data acquisition and analysis were performed using the JPKSPM Data Processing Software (JPK version 4.2.50).

MV3 cells were detached with EDTA (0.02%)/trypsin (0.25% final concentration) and a first batch of them was seeded subconfluently onto a collagen I (0.4 mg/ml collagen diluted in PBS, from bovine calf skin; Biochrom) coated glass bottom dish (FluoroDish FD35–100, World Precision Instruments, Sarasota, USA). They were bathed in HEPES-buffered (20 mmol/L) RPMI medium (Sigma) for different MV3 cell clones or in HEPES-buffered (10 mmol/L) RPMI medium of the desired pH value in the experimental chamber of the AFM at 37 °C. After a minimum of ~60 min a second batch of the detached melanoma cells was added. One of these not yet adherent cells was picked with the CellTak^®^ (cell and tissue adhesive, BD Biosciences, San Jose, USA) coated cantilever (tipless silicon SPM-sensor, 0.03 N/m, Nanoworld, Neuchâtel, Switzerland). The cantilever had been coated in ~8 μl CellTak for 20 min prior to the experiment. Additional control experiments had revealed that the adhesion to CellTak is pH-independent in a range of pH_e_7.4 to pH_e_6.4. Thus, we can assume that the adhesion strength of the cell attached to the cantilever was not affected by changing pH_e_ during the course of our experiments. The cantilever was carefully positioned under optical control above a spherical cell. A maximum loading force of <1 nN and 5–10 s contact time was used to firmly attach an MV3 cell to the front part of the CellTak-coated cantilever which was then lifted. The procedure of the “cell picking” was highly standardized in order to minimize variations in cell geometry and adhesion forces between the CellTak-coated cantilever and the picked cell^49^. Accordingly, the stiffness of the cells attached to the cantilever did not differ between individual cells. Similarly, varying pH_e_ from 7.4 to pH_e_6.4 did not change the stiffness of MV3 cells.

The cell attached to the cantilever was lowered onto an adherent cell on the glass bottom dish with a constant force of 1.5 nN at a cantilever velocity of 5 μm/s during approach/retraction. After a contact time of 2 s, tumour cells were separated by lifting the cantilever. The force needed to fully separate the cells is measured as maximum pulling force and is represented by the minimum value of the retraction curve. The area under the curve represents the required energy to pull the cells apart. It is calculated by the area that is enclosed between the force curve and the x-axis set to baseline^50^. Figure 8b shows representative force-distance curves measured for the same cell pairings at pH_e_7.4 and pH_e_6.4, respectively. The attached cell at the cantilever was used for more measurements on other adherent cells (<15 cells per condition). SCFS was performed in a paired way at varying pH_e_. To this end one immobilised cell at the cantilever was used to probe 10–15 adherent MV3 cells that were consecutively exposed to different pH_e_. An equilibration period of 10 min was allowed after each change of pH_e_. N = cells attached to the cantilever; n = cells on the underlying matrix.

### Multicellular cell aggregation assay

To determine the adhesive potential of MV3 cells in the initial formation of a multicellular spheroid, aggregation assays were performed similarly as described in^23^,^51^. Multi-well plates were coated with a layer of HEPES-buffered medium containing 1% agar and 10% FCS. 250.000 cells/well were cultured in 1 ml medium for 16 h on a shaker (100 rpm, 37 °C). pH dependence was studied by adequately adjusting pH of the bottom layer and the bicarbonate-free RPMI-1640 medium supplemented with HEPES (10 mmol/L). Formation of tumour spheroids was monitored using an Axiovert25 microscope (Zeiss, Oberkochen, Germany) equipped with an AxioCam ICc1 (Zeiss) and a 5 × 0.12 objective. Data acquisition of light microscopy images and analysis of the cross-sectional area of the tumour spheroids was performed with the AxioVision SE64 Rel. 4.9. software. N = individual experiments; n = spheroids.

### Immunofluorescence staining of ALCAM in 2D cultures

MV3 cells were seeded onto glass coverslips coated with collagen I (0.4 mg/ml collagen diluted in PBS, from bovine calf skin; Biochrom). Cells were fixed with 3.5% PFA in PBS for 30 min at RT. Afterwards, cells were washed 3x in PBS, permeabilised with 0.1% Triton X-100 in PBS and again washed 3x in PBS. Blocking occurred in 3% BSA diluted in PBS for 3 h at RT. The monoclonal mouse anti-CD166/ALCAM antibody (L50; dilution 1:100 in 3% BSA-PBS; ThermoFisher Scientific) served as primary antibody incubated at 4 °C overnight. After the next washing procedure, the secondary antibody (goat anti-mouse Alexa 488, dilution 1:600 in 3% BSA-PBS; Molecular Probes, Eugene, USA) was incubated for 45 min at RT and probes were washed again. Fluorescence mounting medium (Dako, Glostrup, Denmark) supplemented with DAPI (Invitrogen) was used to mount the samples. Immunofluorescence images were acquired under wide-field fluorescence conditions with an inverted microscope (Axiovert 200, Zeiss) equipped with a 100 × 1.45 objective lens. The following filter sets were used: excitation 570/40 nm, beam splitter 510 nm, emission 540/50 nm (for Alexa 488-fluorescence) and excitation 365/12 nm, beam splitter 395 nm, emission 397 nm (for DAPI fluorescence). Image acquisition was performed with a digital camera (model 9.0, RT-SE-Spot; Visitron Systems, Puchheim, Germany) and the MetaVue software (Visitron Systems).

### Western Blot analysis of MCAM from spheroid cultures

MV3 spheroids from cell aggregation assays were harvested, washed in PBS twice, lysed in radioimmunoprecipitation assay (RIPA) lysis buffer and further treated as described previously for confluent MV3 monolayer (20 μg protein/sample)^14^. A primary monoclonal rabbit anti-MCAM (CD146) antibody (dilution 1:2.000; Merck Millipore, Billerica, USA) and, as a loading control, a monoclonal mouse anti-β-actin antibody (dilution: 1:10.000, Sigma-Aldrich) were used. Peroxidase-conjugated antibodies served as secondary antibody: IgG anti-rabbit POD (dilution 1:10.000, Merck) for MCAM detection and goat anti-mouse POD (dilution 1:50.000, Dianova, Hamburg, Germany) for β-actin. Quantification of three individual protein isolations was performed in duplicates using Fiji/Image J software (version 1.50 g) and MCAM expression was corrected for β-actin levels.

### Analysis and statistics

Parametric data are expressed as bar diagrams and represent the mean value ± SEM. Statistical significance was tested using one-way ANOVA and, when statistical differences were detected, post hoc comparisons by student’s t-test. Non-parametric data are expressed as box plots which represent the mean (square), median (horizontal line), 25th and 75th percentile (box) and outlier (whiskers). In this case, values are specified as median (Q1, first quartile/Q3, third quartile). Statistical significance was tested with the Kruskal-Wallis ANOVA followed by the Mann-Whitney U test. OriginPro 8 (Origin Lab Corp., Northampton, MA, USA) was used for graphical and statistical exploration of the data. Significance levels are indicated as follows: *p < 0.05, **p < 0.01, ***p < 0.001. # = significant difference (p < 0.001) to all other groups.

## Additional Information

**How to cite this article**: Hofschröer, V. *et al*. Extracellular protonation modulates cell-cell interaction mechanics and tissue invasion in human melanoma cells. *Sci. Rep.* **7**, 42369; doi: 10.1038/srep42369 (2017).

**Publisher's note:** Springer Nature remains neutral with regard to jurisdictional claims in published maps and institutional affiliations.

## Supplementary Material

Supplementary Material
