# Supplementary material for: Extracellular protonation modulates cell-cell interaction mechanics and tissue invasion in human melanoma cells
Source: Sci Rep. 2017 Feb 13;7:42369. doi: 10.1038/srep42369 (PMC5304230; doi:10.1038/srep42369)
Supplement: Supplementary Material [file srep42369-s1.pdf]

SUPPLEMENTARY INFORMATION

Extracellular protonation modulates cell-cell interaction mechanics and tissue invasion in human melanoma cells

Verena Hofschröer[1]*, Alexander Koch[1], Florian Timo Ludwig[1], Peter Friedl[2,3,4], Hans Oberleithner[1], Christian Stock[5#] and Albrecht Schwab[1#]

[1]Institute of Physiology II, University of Münster, Münster, Germany

[2]Radboud University Medical Centre, Radboud Institute for Molecular Life Sciences, Department of Cell Biology, Nijmegen, The Netherlands

[3]David H. Koch Center for Applied Research of Genitourinary Cancers, The University of Texas MD Anderson Cancer Center, Houston, Texas, United States

[4]Cancer Genomics Center,CG Utrecht, The Netherlands

[5]Department of Gastroenterology, Hannover Medical School, Hannover, Germany

[#]authors contributed equally

Figure S1

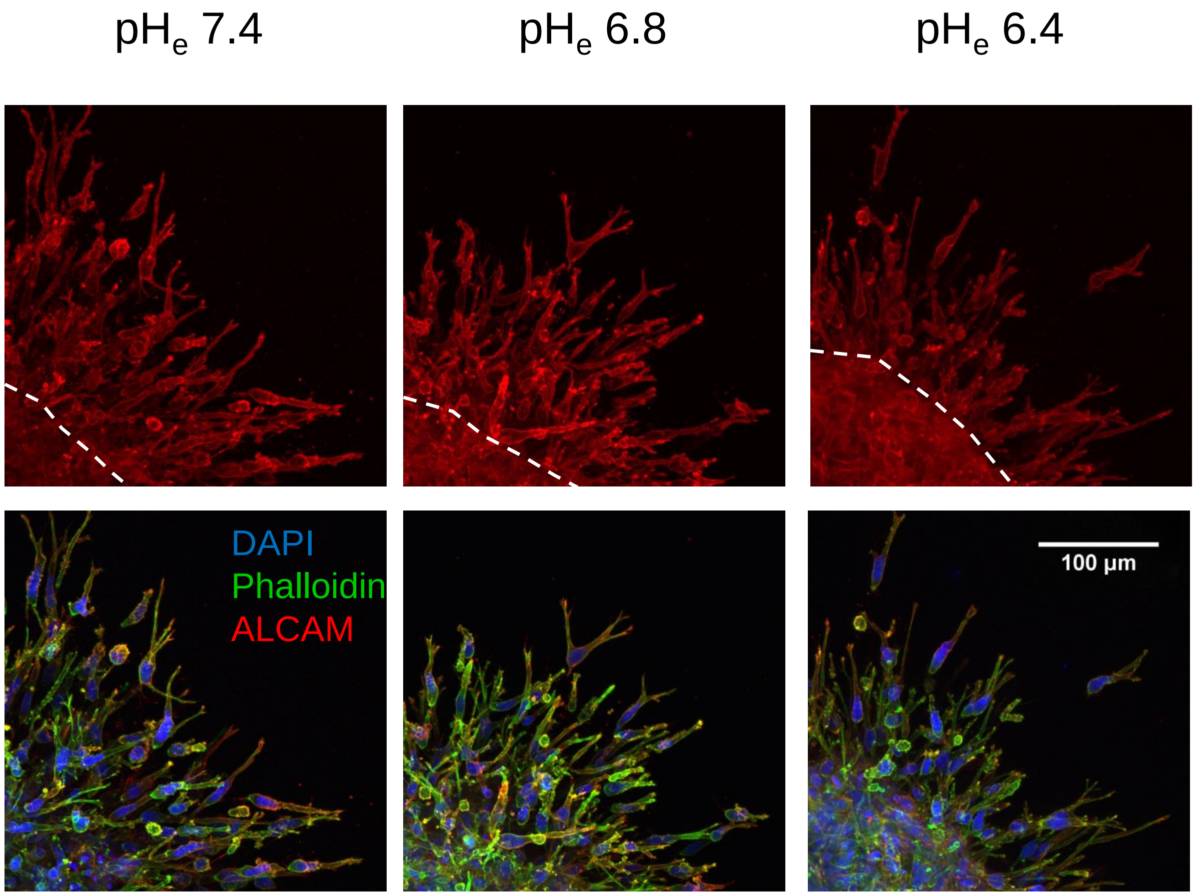

<u>Figure S1</u>: Close-ups of MV3 migration in 3D through a collagen I meshwork at varying pH_e. The dashed line indicates the interphase between spheroid core and invasion zone. Images were obtained with an Olympus FV1000 scanner confocal microscope equipped with a 40x/0.8NA water immersion objective.

Figure S2

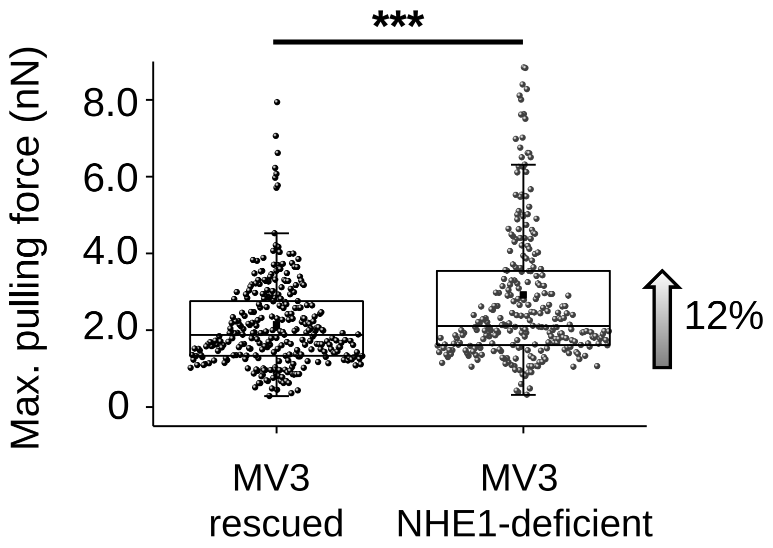

Figure S2: Single cell force spectroscopy using MV3 cells with different NHE1 expression levels. NHE1-deficient MV3 cells showed a significant 12% increase in the maximum pulling force compared to the respective control (NHE1-deficient: 2.11 nN (1.61/3.55 nN, N = 3, n = 286); rescued: 1.88 nN (1.34/2.76 nN, N = 3, n = 299)).

Figure S3

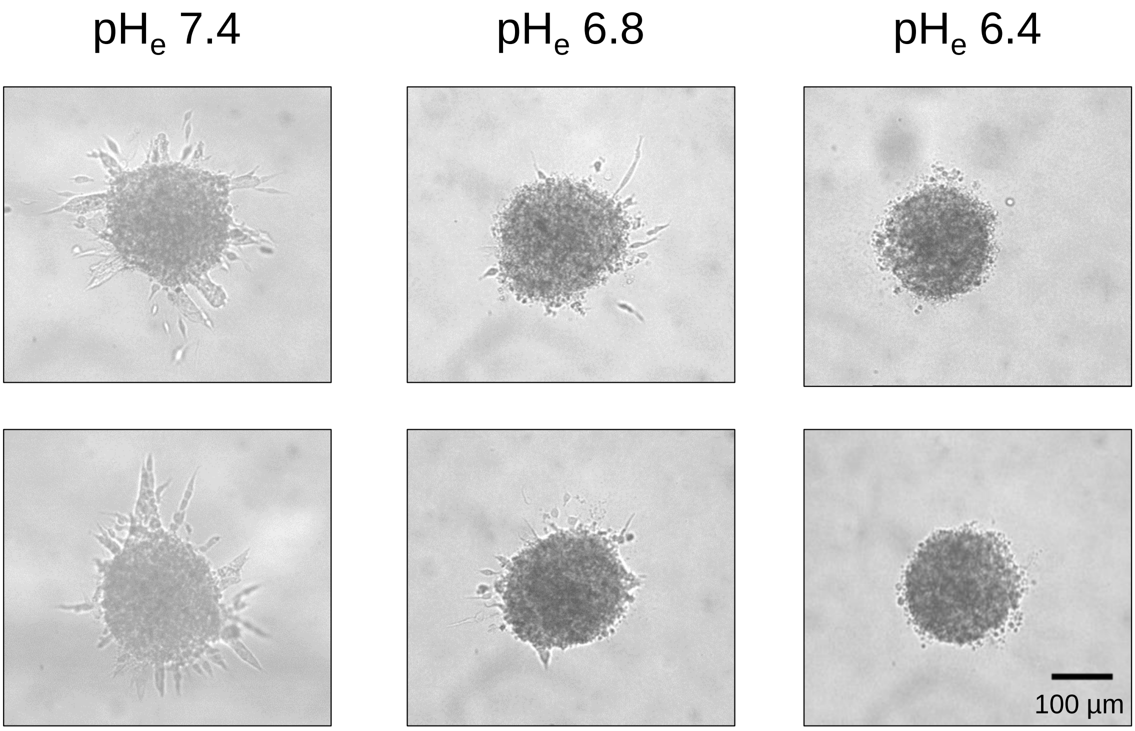

Figure S3: 3D migration of breast cancer cells (4T1; 1.000 cells / spheroid for 24h) through a collagen I (4 mg/ml) meshwork. Increasing the extracellular proton concentration reduces the formation of solid strands and leads to single cell migration.
